# Molecular Regulation of Zn^2+^ Solvation Structure and Interphase Evolution by Glutaronitrile for Stable Aqueous Zinc Metal Batteries

**DOI:** 10.3390/nano16150942

**Published:** 2026-07-30

**Authors:** Zhongyu Wan, Dong Li, Fei Wang, Houzhao Wan

**Affiliations:** Hubei Key Laboratory of Micro-Nanoelectronic Materials and Devices, School of Integrated Circuits, Hubei University, Wuhan 430062, China; 202331127002042@stu.hubu.edu.cn (Z.W.);

**Keywords:** aqueous zinc metal batteries, glutaronitrile, Zn^2+^ solvation structure, ZnF_2_-rich interphase, dendrite suppression

## Abstract

Aqueous zinc metal batteries are promising for safe and cost-effective energy storage. However, their practical application is limited by the intrinsic instability of the Zn/electrolyte interface, including water-induced hydrogen evolution, Zn corrosion, and dendrite-prone Zn deposition. Herein, glutaronitrile (GLN) is introduced as a multifunctional dinitrile additive to stabilize Zn metal anodes through coupled regulation of solvation chemistry and interfacial evolution. The polar C≡N groups of GLN can coordinate with Zn^2+^, to replace part of the water molecules in the primary solvation shell, thereby suppressing the activity of coordinated water. Meanwhile, uncoordinated C≡N groups act as hydrogen-bond acceptors to reorganize the surrounding water network, further suppressing free-water participation in hydrogen evolution and corrosion. This dual regulation optimizes the Zn/electrolyte interfacial environment, improves electrolyte wettability on Zn, homogenizes Zn^2+^ flux, and promotes compact, dendrite-suppressed Zn deposition. Additionally, GLN promotes the formation of a chemically heterogeneous interfacial structure enriched with ZnF_2_ in the inner region, which further protects the Zn surface and stabilizes the Zn plating/stripping process. The optimized ZHG6 electrolyte enables Zn||Zn symmetric cells to cycle stably for over 900 h at 1 mA cm^−2^ and 1 mAh cm^−2^, while Zn||Cu cells maintain high Coulombic efficiency during long-term cycling. Furthermore, Zn||V_6_O_13_ full cells exhibit enhanced cycling stability and rate capability, achieving stable operation for 3200 cycles at 5 A g^−1^. As evidenced in this work, dinitrile-based molecular additives provide an effective and scalable strategy to fabricate durable aqueous zinc metal batteries.

## 1. Introduction

Recently, aqueous zinc-ion batteries (AZIBs) have been regarded as one of the most promising emerging energy-storage systems owing to their high safety, low cost, and abundant resource availability, making them particularly attractive for grid-scale energy storage and large-scale low-cost applications [1,2,3,4,5]. However, zinc metal anodes are not thermodynamically stable in aqueous electrolytes. In conventional electrolyte environments, Zn^2+^ generally exists in the form of [Zn(H_2_O)_6_]^2+^, and its desolvation process is kinetically sluggish. Meanwhile, highly active water molecules readily trigger the hydrogen evolution reaction (HER) [6,7], zinc corrosion, and the formation of basic by-products [8,9]. In addition, the non-uniform deposition of Zn^2+^ on the electrode surface further induces zinc dendrite growth [10,11,12,13], leading to local electric-field distortion, accumulation of inactive “dead Zn”, and even internal short circuits [14,15,16]. Therefore, the structural and interfacial instability of the Zn anode/electrolyte interface has become a critical bottleneck limiting the cycling life, Coulombic efficiency (CE), and practical implementation of AZIBs [17].

To address these challenges, extensive efforts have been devoted to electrolyte optimization [18], interfacial coating construction [19], separator modification [20], and electrode-material design [21]. Among these strategies, regulating the solvent composition of the electrolyte can effectively alter the solvation structure of Zn^2+^ [22,23], thereby improving the reversibility of zinc plating/stripping and stabilizing interfacial reaction kinetics [24,25]. On this basis, electrolyte additives have attracted widespread attention because of their cost-effectiveness, process scalability, and precise interfacial regulation capability. This strategy can reconstruct the Zn^2+^ solvation structure or induce the in situ formation of interfacial layers, thereby promoting uniform nucleation behavior and significantly enhancing the stability of zinc anodes [26]. For instance, Ni et al. [27] introduced glycerol as a co-solvent, which formed a strong hydrogen-bonding network with water molecules, effectively weakening the Zn^2+^ solvation structure and improving the stability of low-temperature electrolytes as well as the reversibility of zinc plating/stripping. Zhang et al. [28] employed potassium hexafluorophosphate as an additive to construct an in situ ZnF_2_/ZnS-rich composite solid electrolyte interphase (SEI) layer on the zinc anode surface, effectively suppressing dendrite growth, hydrogen evolution, and electrochemical corrosion, thereby markedly improving interfacial stability [29]. These studies demonstrate that both solvation-structure regulation and SEI construction can effectively stabilize zinc metal anodes. Nevertheless, most reported additives mainly regulate one or two of these processes, whereas establishing a continuous molecular regulation pathway linking solvation chemistry, water-network evolution, and interphase formation remains challenging.

Therefore, designing small-molecule additives with well-defined coordination sites that can simultaneously regulate the solvation structure [30] and interfacial film-forming behavior [31] is of great significance for further improving the overall performance of AZIBs. In this work, glutaronitrile (GLN, NC(CH_2_)_3_CN), an organic nitrile small molecule with a dicyano structure, is selected as an electrolyte additive to synergistically enhance the stability of zinc anodes from the perspectives of molecular coordination regulation and interfacial-structure construction. The cyano groups (-C≡N) in glutaronitrile possess strong polarity and coordination capability, enabling competitive coordination with Zn^2+^ and partial substitution of water molecules in the primary solvation sheath. This weakens the parasitic corrosion of zinc anodes caused by active water and optimizes the Zn^2+^ desolvation pathway and deposition kinetics. Meanwhile, glutaronitrile can induce the formation of a gradient SEI at the electrode/electrolyte interface. The outer organic components of this SEI effectively block direct corrosion by the electrolyte, whereas the inner ZnF_2_-rich inorganic components provide efficient Zn^2+^ transport channels [32,33]. The optimized ZHG6 electrolyte enables Zn||Zn symmetric cells to cycle stably for over 900 h at 1 mA cm^−2^ and 1 mAh cm^−2^, while Zn||Cu cells maintain high CE. The GLN adding strategy proposed in this study significantly enhances the interfacial stability of zinc anode. This strategy provides technical support for developing highly stable aqueous zinc-ion batteries.

## 2. Materials and Methods

### 2.1. Materials

In the experiment, all reagents are used directly without any further purification. The relevant parameters of the adopted raw materials are listed as follows: V_2_O_5_ (purity ≥ 99%, Aladdin, Shanghai, China), Zn (CF_3_SO_3_)_2_ (purity ≥ 99%, Adamas-beta, Shanghai, China), C_5_H_6_N_2_ (purity ≥ 98%, Macklin, Shanghai, China), Zn foil (3N purity, Sinopharm Chemical Reagent Co. Ltd., Shanghai, China); conductive agent acetylene black, binder polyvinylidene fluoride (PVDF), and solvent N-methyl-2-pyrrolidone (NMP) are purchased directly from commercial sources; the employed deionized water was lab-made. A glass fiber separator was used in the cell assembly.

### 2.2. Synthesis of V_6_O_13_

V_6_O_13_ cathode material was synthesized via the hydrothermal reaction using V_2_O_5_ as the vanadium precursor. The specific steps are as follows: Firstly, 3.6 g of V_2_O_5_ was dispersed in 40 mL anhydrous ethanol under magnetic stirring, followed by addition of 120 mL of deionized water. The mixture was continuously stirred at 25 °C for 1 h to obtain a homogeneous suspension. Secondly, the homogeneous suspension was transferred to a Teflon-lined stainless-steel autoclave for hydrothermal reaction at 180 °C for 24 h. Finally, after the reaction was cooled to room temperature, the precipitate was collected, fully washed repeatedly with anhydrous ethanol, and vacuum-dried at 75 °C for 12 h to yield V_6_O_13_ powder.

### 2.3. Preparation of the Electrolyte

The base electrolyte employed in this experiment was prepared using 10 mL of Zn(CF_3_SO_3_)_2_ aqueous solution (2 mol L^−1^), which is denoted as ZH. Then, different volume fractions of GLN were added into the base electrolyte under magnetic stirring. The GLN contents were set as 2%, 4%, 6%, 8%, and 10%. The obtained electrolytes separately were labeled ZHG2, ZHG4, ZHG6, ZHG8, and ZHG10. For the matched-control ^1^H NMR measurement, a Zn-free aqueous solution containing 6 vol% GLN was prepared and denoted as GLN6.

### 2.4. Preparation of the Cathode

The V_6_O_13_ powder prepared by the hydrothermal method, acetylene black, and PVDF were mixed at a mass ratio of 7:2:1. The mixture was then completely ground. An agate mortar was used for this step. Next, N-methyl-2-pyrrolidone (NMP) was added drop by drop. This was done to tune the slurry viscosity. A homogeneous electrode slurry was finally obtained. The slurry was uniformly coated onto a 600-mesh stainless-steel mesh. The coated electrode was dried before further use. The stainless-steel mesh was purchased from Biling (Tokyo, Japan). The dried electrode sheet was then punched into circular disks with a diameter of 1.13 cm. The active-material loading of the V_6_O_13_ cathode was approximately 2–3 mg cm^−2^, which was determined by the weighing method. All specific capacities reported in this work were calculated based on the mass of the active material.

### 2.5. Materials and Interphase Characterization

The morphologies of V_6_O_13_ and cycled Zn electrodes were examined by field-emission scanning electron microscopy (FESEM). Energy-dispersive X-ray spectroscopy (EDS) was used to map elemental distributions across the cycled Zn surface layer. X-ray diffraction (XRD) was used to identify the crystal structure of V_6_O_13_ and crystalline phases or by-products formed on Zn after cycling. Electrolyte structure was examined by ^1^H nuclear magnetic resonance (^1^H NMR; D_2_O solvent), Raman spectroscopy (532 nm excitation). Atomic force microscopy (AFM) was used to evaluate the roughness of deposited Zn, and in situ optical microscopy was used to monitor Zn growth during deposition. X-ray photoelectron spectroscopy (XPS; Al Kα) with Ar^+^ sputtering was used to compare the surface and sputtered compositions of cycled Zn electrodes.

### 2.6. Electrochemical Measurements

CR2032 coin cells were assembled for electrochemical measurements using the prepared V_6_O_13_ electrode as the cathode, commercial Zn foil as the anode, and a glass-fiber membrane as the separator. All electrochemical tests were conducted under constant-temperature conditions. Cyclic voltammetry (CV), Tafel polarization, and linear sweep voltammetry (LSV) measurements were performed using a CHI760E electrochemical workstation. Long-term cycling and rate-performance tests were carried out on a Neware eight-channel battery testing system. Zn||Zn symmetric cells were used to evaluate Zn plating/stripping stability, whereas Zn||Cu cells were used to determine the CE. For Zn||Cu cells, a Cu foil (50 μm thick and 1.13 cm in diameter) was used as the working electrode, while a Zn foil served as the counter/reference electrode. Zn plating/stripping was conducted at 1 mA cm^−2^ with a fixed areal capacity of 0.5 mAh cm^−2^. Zn||Zn cells were cycled at 1 mA cm^−2^ with a fixed areal capacity of 1 mAh cm^−2^. For Zn||V_6_O_13_ full cells, CV was conducted between 0.2 V and 1.6 V, and galvanostatic cycling was evaluated at 1 A g^−1^, 3 A g^−1^, and 5 A g^−1^. The ionic conductivities and Zn^2+^ transference numbers of the electrolytes were measured by electrochemical impedance spectroscopy and chronoamperometry combined with impedance measurements, respectively.

## 3. Results

### 3.1. Design Rationale for GLN-Regulated Zn Deposition

**Figure 1:**
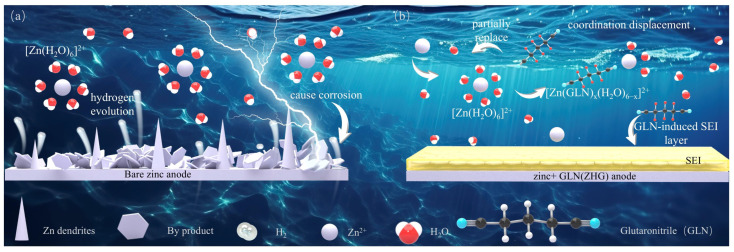
Schematic illustration of the mechanism by which GLN regulates Zn deposition: (**a**) In the base electrolyte, Zn deposition is uneven. Electrode suffers from severe corrosion. (**b**) In the GLN-modified electrolyte, GLN regulates the Zn^2+^ solvation structure. It also induces the formation of a stable SEI layer. These effects promote uniform Zn deposition and suppress side reactions.

The proposed working mechanism is illustrated in Figure 1. As shown in Figure 1a, in the baseline aqueous electrolyte, the Zn surface is directly exposed to hydrated Zn^2+^ species and electrochemically active water, which can induce water reduction [34], Zn corrosion, and non-uniform deposition. These processes lead to surface roughening and may further promote dendrite growth and internal short-circuit failure [35].

As shown in Figure 1b, the GLN molecule contains two polar cyano groups. This molecular structure can regulate the local coordination environment of Zn^2+^. It can also affect the hydrogen-bond network among water molecules. During the cycling process, GLN may further control the interfacial conversion behavior of electrolyte components on the Zn surface. This process can induce the formation of a stable SEI layer. This mechanism points to a synergy. It combines bulk electrolyte tuning with interfacial stabilization. GLN can suppress water-related side reactions by adjusting the electrolyte microenvironment. Meanwhile, the interfacial layer induced by GLN can reduce the continuous consumption of the electrolyte. It can also improve the stability of the Zn anode [36].

### 3.2. Optimization of GLN Content and Electrolyte Microenvironment

To screen the optimal amount of glutaronitrile, we assembled Zn||Zn symmetric cells with electrolytes containing different concentrations of glutaronitrile. The cells were cycled for 20 cycles at 1 mA cm^−2^ and 1 mAh cm^−2^. In this work, the cells were disassembled after cycling. The zinc anodes were then rinsed with anhydrous ethanol to remove residual electrolyte and separator fragments from their surfaces. The zinc anodes were further characterized by SEM. As shown in Figure S1, the zinc anode in the ZH group shows the most obvious dendrite growth. After glutaronitrile was added, the morphology of zinc deposition was improved. Among these samples, the ZHG6 group shows a relatively flat and compact surface. This result indicates that 6% glutaronitrile can effectively suppress dendrite growth. When the volume fraction of glutaronitrile was further increased to 8% and 10%, the improvement in surface morphology became weaker. In general, 6% glutaronitrile can be preliminarily identified as the optimal additive ratio.

**Figure 2:**
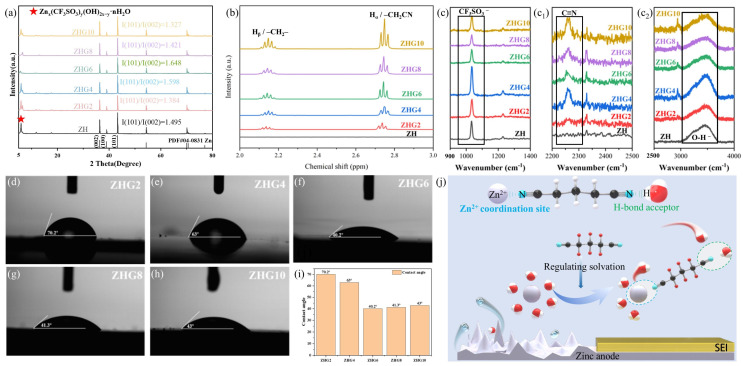
(**a**) XRD patterns of Zn anodes after cycling; (**b**) ^1^H NMR spectra of the electrolytes; (**c**,**c_1_**,**c_2_**) Raman spectra in the ranges of 900–1400 cm^−1^, 2200–2500 cm^−1^, and 2500–4000 cm^−1^, respectively; (**d**–**h**) contact-angle images of the GLN-containing electrolytes on Zn foil; (**i**) summary of the contact-angle variation; (**j**) proposed mechanism for ZHG-mediated regulation of the Zn anode interface.

Under the same experimental conditions, XRD characterization was carried out on the cycled Zn anode sheets. This test was used to analyze the formation of by-products. As presented in Figure 2a, all samples exhibited typical diffraction peaks of metallic Zn within the 2θ range of 35–45°. This result was confirmed by comparison with the standard Zn card (PDF# 04-0831). These diffraction peaks correspond to the (100), (002), and (101) planes of Zn. In the low-angle region of 2θ = 5–10°, the ZH sample showed an obvious diffraction peak related to by-products. This peak can be assigned to basic zinc trifluoromethanesulfonate by-products, Zn_x_(CF_3_SO_3_)_y_(OH)_2x−y_·nH_2_O [37]. As the glutaronitrile content increased to 6%, this by-product peak became much weaker. This result indicates that an appropriate amount of glutaronitrile can suppress side reactions between the electrolyte and the Zn anode. When the volume fraction of glutaronitrile was further increased to 8% and 10%, the by-product peak became stronger again. The crystallographic orientation of Zn was further evaluated using the integrated intensity ratio of the Zn(101) and Zn(002) reflections [38]. The I(101)/I(002) ratios were 1.495, 1.384, 1.598, 1.648, 1.421, and 1.327 for ZH, ZHG2, ZHG4, ZHG6, ZHG8, and ZHG10, respectively. ZHG6 exhibited the highest ratio, indicating a relatively enhanced Zn(101) orientation compared with Zn(002). This result suggests that excessive additive may weaken the interfacial stabilization effect and lead to increased by-product formation. SEM observations and XRD patterns offered consistent evidence. The ZHG6 system was more effective at inhibiting dendrites. It also reduced unwanted by-products more efficiently. These results further support the selection of ZHG6.

As shown in Figure 2b, the ZH electrolyte shows almost no obvious proton resonance signals in the range of 2.0–3.0 ppm. After glutaronitrile was added, two new ^1^H NMR signals appeared in the spectra. A signal is observed at roughly 2.1–2.2 ppm. This can be ascribed to the central methylene protons (H_β_, -CH_2_-). Another signal is found at about 2.7–2.8 ppm. This is attributed to the methylene protons next to the cyano group (H_α_, -CH_2_CN). As the glutaronitrile content increased, the intensities of the two signals gradually became stronger. This concentration-dependent increase in signal intensity mainly originates from the increasing GLN content and is therefore not used as the criterion for selecting the optimal additive concentration. In contrast, the peaks showed a slight downfield shift. This result indicates that the local electronic environment of the methylene protons changed. This change may be related to the coordination interaction between the cyano group and Zn^2+^, as well as the O-H/O-D···N≡C hydrogen-bonding interaction [39]. These results suggest that glutaronitrile can participate in regulating the solvation structure and hydrogen-bond network of the electrolyte. Considering the above results, an appropriate amount of glutaronitrile helps suppress water-related side reactions. It also improves the reversibility of Zn deposition/stripping. To separate Zn^2+^–GLN coordination from GLN–H_2_O hydrogen bonding, GLN6 and ZHG6 were compared by ^1^H NMR at the same GLN concentration. As shown in Figure S2, the GLN methylene signals shifted from 2.569 and 1.966 ppm in GLN6 to 2.664 and 2.062 ppm in ZHG6. Both signals moved downfield after the addition of Zn^2+^. This change indicates that Zn^2+^ alters the local chemical environment of GLN. It also supports the involvement of GLN in the local Zn^2+^ coordination structure. The water-proton region was also examined. The signals of ZH, GLN6, and ZHG6 appeared at 4.667, 4.701, and 4.744 ppm, respectively (Figure S3). These shifts indicate a change in the average water environment. They also suggest that GLN affects the hydrogen-bond network of water.

Raman spectroscopy was used to study the effect of GLN on the Zn^2+^ coordination environment and the hydrogen-bond network. Figure 2(c,c_1_,c_2_) show the main Raman features. The bands between 900 and 1400 cm^−1^ arise from the S-O and C-F vibrations of CF_3_SO_3_^−^. The ZHGx electrolytes show an additional band between 2200 and 2500 cm^−1^. This band corresponds to the C≡N stretching vibration and confirms the presence of GLN in the electrolyte. The C≡N band shows small changes among the samples. These changes suggest that the local environment of the nitrile groups is altered. The interaction between GLN and Zn^2+^ may cause these changes. The broad O-H band between 3000 and 4000 cm^−1^ also changes slightly. This result suggests a change in the average water environment. However, the Raman changes are small. Therefore, the Raman spectra are used only as supporting evidence. Together with the matched-control ^1^H NMR results, these findings support the interaction between GLN and Zn^2+^. They also suggest that GLN changes the hydrogen-bond network of water.

Contact angle measurements were used to evaluate the wetting behavior of different electrolytes on the zinc metal surface. As shown in Figure 2d–h, the wettability of the electrolyte on zinc foil was significantly improved after adding an appropriate amount of glutaronitrile (GLN). When the GLN content increased from 2% to 6%, the contact angle gradually decreased from 70.2° for ZHG2 to 40.2° for ZHG6 (Figure 2i). This result suggests that a moderate GLN addition is beneficial. It enhances the compatibility at the electrolyte/Zn interface. This behavior may be related to the interaction between the C≡N group and the zinc surface. Good wettability helps the electrolyte spread more uniformly on the zinc surface. This leads to a more homogeneous Zn^2+^ flux distribution. It also provides favorable conditions for uniform deposition. When the GLN content was further increased, the contact angles of ZHG8 and ZHG10 respectively increased to 41.3° and 43.0°. This suggests that excessive additive does not further improve wettability. This may be because excessive GLN changes the polarity and surface tension of the electrolyte. As a result, the spreading ability on the zinc surface is slightly reduced [40]. Therefore, ZHG6 shows the best wetting performance. This result is also consistent with the SEM and XRD analyses. In those analyses, ZHG6 showed strong suppression of dendrite growth and side-product formation. The ionic conductivities of the electrolytes with different GLN contents are shown in Figure S4. The conductivity gradually decreases as the GLN content increases. It drops from 86.49 mS cm^−1^ for ZH to 81.60 mS cm^−1^ for ZHG6. A further decrease to 73.80 mS cm^−1^ is observed for ZHG10. Figure S5 presents the measured Zn^2+^ transference numbers. The value rises from 0.17 for ZH to a maximum of 0.61 for ZHG6. It then declines to 0.41 for ZHG8 and 0.31 for ZHG10. Overall, ZHG6 provides the most favorable balance between ionic conductivity and selective Zn^2+^ transport.

According to these findings, Figure 2j presents the possible mechanism through which ZHG modulates the Zn anode interface. The C≡N functional groups in GLN can interact with Zn^2+^ through coordination. This interaction allows GLN to participate in the local coordination environment of Zn^2+^ and modify the average Zn^2+^ solvation structure. Meanwhile, C≡N groups that are not fully involved in Zn^2+^ coordination can serve as hydrogen-bond acceptors to regulate the spatial distribution and activity of surrounding water molecules. This dual function reduces the possibility of free water participating in hydrogen evolution and corrosion reactions, while improving the wettability of the Zn anode interface and promoting a more uniform Zn^2+^ flux and Zn deposition behavior. Consequently, ZHG6 effectively stabilizes the Zn anode interface through the synergistic effects of solvation-sheath reconstruction, hydrogen-bond-network regulation, and interfacial-wettability optimization [41].

### 3.3. Morphology Evolution and Dendrite-Suppressed Zn Deposition

**Figure 3:**
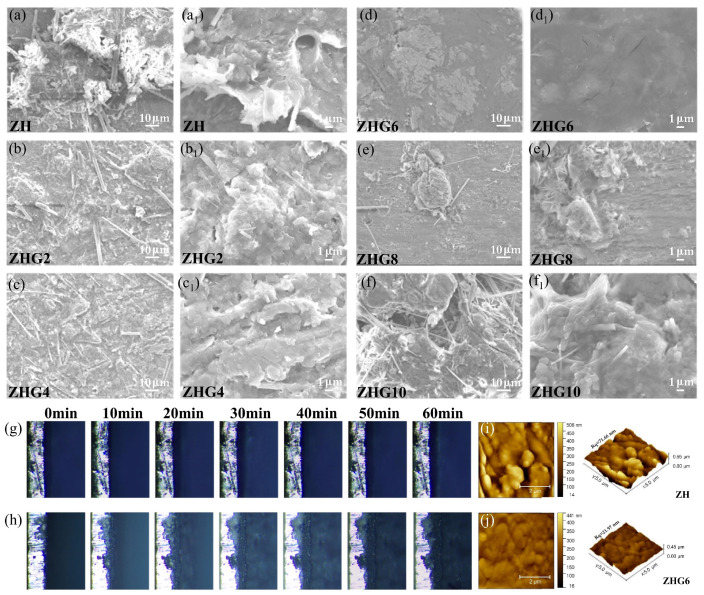
SEM images of Zn electrodes harvested from Zn||Zn symmetric cells after 50 cycles at 1 mA cm^−2^ and 1 mAh cm^−2^: (**a**,**a_1_**) ZH; (**b**,**b_1_**) ZHG2; (**c**,**c_1_**) ZHG4; (**d**,**d_1_**) ZHG6; (**e**,**e_1_**) ZHG8; (**f**,**f_1_**) ZHG10; (**g**,**h**) optical microscopy images showing the interfacial evolution of ZHG6 and ZH during Zn deposition for 0–60 min; (**i**,**j**) AFM height images and 3D topographic profiles of Zn deposited in ZH and ZHG6, respectively.

To evaluate the effect of different electrolytes on the morphology of zinc deposition, Zn||Zn symmetric cells were cycled for 50 cycles at 1 mA cm^−2^ and 1 mAh cm^−2^. The Zn anodes were then characterized by SEM, as shown in Figure 3(a–f,a_1_,b_1_,c_1_,d_1_,e_1_,f_1_). In the ZH electrolyte, the Zn anode showed a rough surface. It also exhibited obvious uneven deposition and dendrite growth (Figure 3(a,a_1_)). After a suitable amount of glutaronitrile was added, the surface flatness of the Zn anode was clearly improved. Among all samples, the ZHG6 electrolyte produced a more uniform and compact deposition morphology. This result indicates that ZHG6 effectively regulates Zn^2+^ deposition behavior and suppresses dendrite growth (Figure 3(d,d_1_)). Nevertheless, further increasing the GLN content to 8% and 10% led to renewed surface degradation. The Zn anode exhibited uneven deposition in localized areas. Aggregated particles were clearly visible. Dendrite-like formations were also found on the surface once more. The surface flatness also decreased to some extent (Figure 3(e,e_1_,f,f_1_)). These results suggest that excessive glutaronitrile may be unfavorable for Zn^2+^ transport and uniform zinc deposition.

In situ optical microscopy was used to observe the dynamic changes during the zinc deposition process. As shown in Figure 3h, zinc deposition in the ZH electrolyte was clearly uneven. The deposition interface gradually became rough, and dendrite-like protrusions appeared during the process. In contrast, the zinc deposition interface in the ZHG6 electrolyte was much smoother, as shown in Figure 3g. During the 60 min deposition process, the interface showed no obvious dendrite growth. This outcome is indicative of the importance of the ZHG6 electrolyte. It encourages uniform Zn deposition across the electrode surface. It also prevents the formation of dendrites to a significant extent.

AFM analysis further confirms this conclusion at a smaller length scale. Compared with the rough and highly fluctuating Zn surface formed in ZH, the ZHG6 surface is smoother and denser, with the root-mean-square roughness decreasing from 71.66 nm for ZH (Figure 3i) to 21.97 nm for ZHG6 (Figure 3j). This pronounced reduction demonstrates that ZHG6 suppresses not only micrometer-scale dendrites and local protrusions but also nanoscale height fluctuations of the Zn deposition surface. Collectively, the SEM, in situ optical microscopy, and AFM observations demonstrate that an appropriate GLN concentration improves Zn nucleation and growth behavior, with ZHG6 showing the strongest suppression of Zn deposition-front roughening.

### 3.4. Electrochemical Stability and Zn Plating/Stripping Reversibility

**Figure 4:**
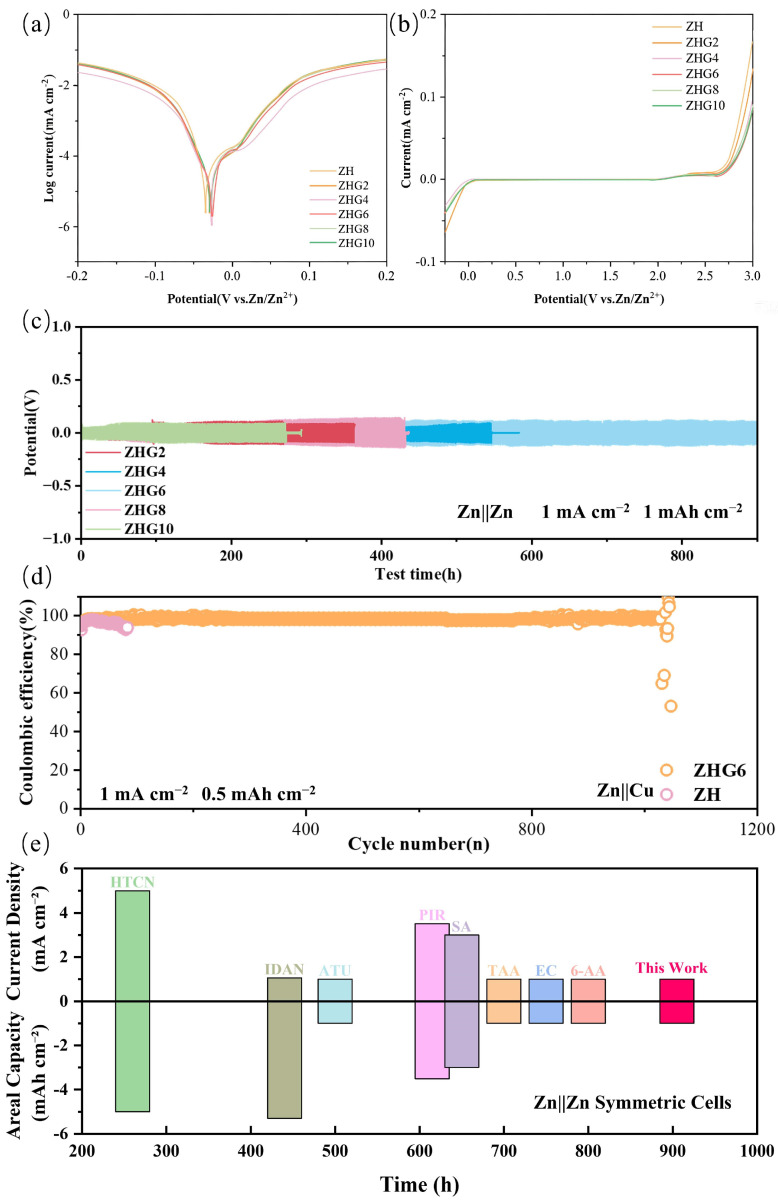
Electrochemical performance of Zn anodes in the indicated electrolytes: (**a**) Tafel polarization curves of Zn in the indicated electrolytes; (**b**) linear sweep voltammetry curves of different electrolytes; (**c**) long-term Zn||Zn cycling at 1 mA cm^−2^ and 1 mAh cm^−2^; (**d**) Coulombic efficiency of Zn||Cu cells; (**e**) comparison of Zn||Zn cycling performance for representative polar electrolyte additives.

To evaluate the effect of the glutaronitrile additive on the corrosion behavior of the Zn anode, Tafel tests were carried out in different electrolytes. The results are shown in Figure 4a. Without glutaronitrile, the corrosion potential of the Zn electrode is about −0.04 V. After the addition of glutaronitrile, the corrosion potential shifts slightly in the positive direction. The corrosion current density also decreases. These changes indicate that glutaronitrile can suppress Zn anode corrosion. Among the tested systems, the ZHG6 system shows a corrosion potential of about −0.03 V and a relatively low corrosion current density. This finding implies that the ZHG6 system exhibits favorable anti-corrosion properties.

LSV tests were used to evaluate the electrochemical stability of different electrolytes. As shown in Figure 4b, the ZH electrolyte shows a more obvious increase in current at high potentials. This result indicates that the ZH electrolyte is more likely to undergo side-reaction-related electrochemical processes. After GLN is added, the current response at high potentials decreases overall. This change suggests that GLN can improve the stability of the electrolyte and suppress unwanted side reactions. Among these electrolytes, the ZHG6 system shows a relatively low current response. This outcome suggests that the ZHG6 system has a lower side-reaction-related current response. These observations are in good agreement with the morphological findings and the Tafel measurements. Taken together, they further imply that a suitable dosage of GLN can alleviate interfacial instability. It also facilitates more uniform zinc deposition.

As shown in Figure 4c, this study tested Zn||Zn symmetric cells with different electrolytes under 1 mA cm^−2^ and 1 mAh cm^−2^. The results show that the ZHG6 electrolyte provides better cycling stability. When the glutaronitrile content increased from 2% to 6%, the cycling life of the symmetric cell increased from about 270 h to more than 900 h. During the whole cycling process, the ZHG6 cell showed no obvious short circuit. It also showed no sudden voltage fluctuation related to cell failure. At the same time, the ZHG6 cell showed a lower polarization voltage. This result indicates that this electrolyte can make the Zn plating/stripping process more stable. It can also improve the reaction kinetics at the electrode interface. By contrast, the ZHG8 and ZHG10 cells did not show further improvement in cycling performance. Their ionic conductivities decrease to 76.6 and 73.8 mS cm^−1^, while their Zn^2+^ transference numbers decrease to 0.41 and 0.31, respectively, compared with 0.61 for ZHG6 (Figures S4 and S5). These results indicate that ZHG6 achieves the optimal balance between ionic conductivity and selective Zn^2+^ transport, whereas further increasing the GLN content compromises ion-transport characteristics and thus limits the reversibility of Zn plating/stripping.

Zn||Cu half-cells were assembled to further evaluate the reversibility of the zinc plating/stripping process, as shown in Figure 4d. In the ZH electrolyte, the Coulombic efficiency decreased obviously after a limited number of cycles. This result indicates that severe side reactions and irreversible zinc consumption occurred in this system. In contrast, the Zn||Cu half-cell using the ZHG6 electrolyte maintained a Coulombic efficiency above 97% during long-term cycling. It also operated stably for about 1000 cycles. These observations suggest that an optimized GLN concentration offers clear advantages. It considerably boosts the reversible behavior of zinc plating and stripping. It also fortifies the electrode interface against degradation.

Figure 4e and Table S1 compare GLN with several representative polar additives that use similar strategies for electrolyte and interface regulation [42,43,44,45,46,47,48,49]. Under the same testing conditions of 1 mA cm^−2^ and 1 mAh cm^−2^, ZHG6 achieves a cycling life of more than 900 h. This value is higher than those of ATU (500 h), 6-AA (800 h), and EC (750 h) [43,44,45]. These results show that GLN offers clear advantages in stabilizing Zn cycling. Its flexible dinitrile structure can coordinate with Zn^2+^ and regulate the water network. These effects also guide the later formation and evolution of the interphase.

### 3.5. Depth-Resolved Surface Chemistry of the Cycled Zn Interphase

**Figure 5:**
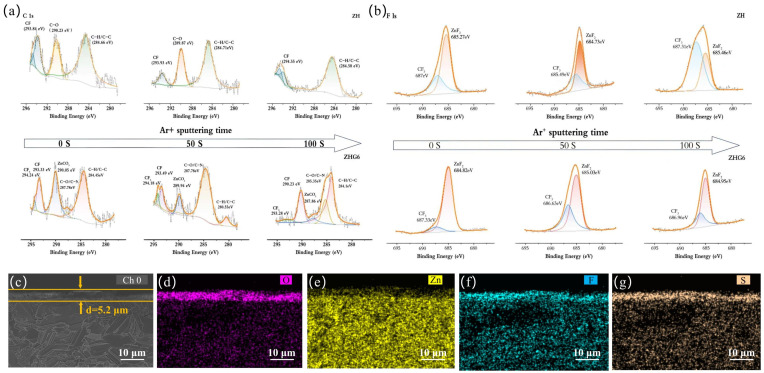
(**a**) XPS C 1s spectra of Zn anodes after cycling in ZH and ZHG6 electrolytes, obtained after Ar^+^ sputtering; (**b**) XPS F 1s spectra of Zn anodes after cycling in ZH and ZHG6 electrolytes, obtained after Ar^+^ sputtering; (**c**) cross-sectional SEM image of the Zn electrode after 50 cycles in the ZHG6 electrolyte; (**d**–**g**) corresponding EDS elemental mapping images.

To analyze the interfacial chemical composition of the Zn anode after cycling, Ar^+^ sputtering depth-profile XPS analysis was carried out on Zn electrodes. These electrodes were cycled for 20 cycles at 1 mA cm^−2^ and 1 mAh cm^−2^. As shown in Figure 5a, the C 1s spectra of both ZH and ZHG6 systems show peaks related to C-C/C-H, C-O, and O-C=O before sputtering. This result indicates that organic residues and electrolyte-derived decomposition products exist on the cycled Zn anode surface. Compared with the ZH system, the ZHG6 system shows an additional signal at around 287.7 eV. This peak may be related to nitrogen-containing carbon species, such as C-N, C≡N, or metal-coordinated carbon species. This result suggests that GLN may participate in the formation of the interfacial layer on the Zn anode surface.

As shown in Figure 5b, the F 1s spectra further reveal the changes in fluorine-containing species with increasing sputtering time. Before sputtering, both ZH and ZHG6 systems show peaks related to -CF_3_ and Zn-F/ZnF_2_. These peaks correspond to triflate residues or their decomposition fragments and inorganic ZnF_2_ species, respectively. Compared with the ZH system, the ZHG6 system shows a slightly stronger ZnF_2_ peak. This result indicates that the addition of GLN may promote the conversion of fluorine-containing species into ZnF_2_ at the interface.

As the Ar^+^ sputtering time increases, surface adsorbates and part of the organic components are gradually removed. The inorganic components inside the SEI layer then become more visible. After sputtering for 50 s, the ZnF_2_-related peak in the F 1s spectrum still shows a strong signal. This result indicates that ZnF_2_ is an important inorganic component inside the interfacial layer. After further sputtering for 100 s, the C 1s spectrum still shows signals related to C–C/C–H, C-O/C-N, and fluorinated carbon species (Figure 5a). This result suggests that a small amount of organic residues or electrolyte decomposition products remains in the deeper interfacial region. In the F 1s spectrum, the ZnF_2_ peak becomes the dominant signal. A certain –CF_3_-related signal is also retained (Figure 5b). These results indicate that this region is mainly composed of inorganic fluorine-containing components, together with a small amount of fluorinated organic residues. Overall, the ZHG6 electrolyte helps construct a stable ZnF_2_-containing interfacial layer on the Zn anode surface. This layer improves interfacial stability and suppresses side reactions.

To investigate the microstructure and chemical composition of the SEI layer, the Zn anode was retrieved after 50 cycles in the ZHG6 electrolyte. It was then fractured in a liquid nitrogen environment to expose its cross-section. The cross-sectional morphology was observed using SEM. The fractured cross-section revealed a dense and uniformly thick SEI layer. Its thickness was measured to be approximately 5.2 μm, as shown in Figure 5c. Further EDS mapping analysis was conducted to examine the elemental distribution. The results indicated that the SEI layer was primarily composed of O, F, and S, with a minor amount of Zn also present (Figure 5d–g). These observations suggest that the addition of glutaronitrile facilitates the formation of a relatively complete SEI layer on the Zn anode surface. For comparison, the cross-sectional SEM image and corresponding EDS elemental mapping of the Zn electrode cycled in the ZH electrolyte are provided in Figure S6. Compared with ZHG6, the ZH electrode exhibits a less uniform interfacial layer, indicating that the protective SEI formed in the absence of GLN is less compact. This comparison further confirms the advantage of the GLN-induced SEI in stabilizing the Zn/electrolyte interface.

These results suggest that the SEI layer formed in the ZHG6 system contains both organic components and fluorine-containing inorganic species. This compact SEI layer acts as a protective interfacial barrier. It reduces direct contact between the electrolyte and the Zn anode. It also helps suppress corrosion and the hydrogen evolution reaction. In addition, the ZnF_2_-rich interfacial layer may guide more uniform Zn^2+^ deposition and stripping. Hence, this stable interfacial structure provides important support for improving the stability of the Zn anode in the ZHG6 system.

### 3.6. Zn||V_6_O_13_ Full-Cell Performance

For the V_6_O_13_ cathode material used in the full cell, its phase composition and morphology first need to be characterized. This step helps confirm whether the hydrothermally synthesized product is the target material. The scanning electron microscopy (SEM) images show that the synthesized powder has a typical layered structure formed by nanoscale stacking (Figure S7). This morphology is highly consistent with the characteristic structure of V_6_O_13_ materials. The X-ray diffraction (XRD) pattern further supports this result (Figure S8). All diffraction peaks of the obtained sample were compared with the standard card of V_6_O_13_ (JCPDS No. 27-1318). The main diffraction peaks of the sample are basically consistent with those in the standard card. Based on the SEM and XRD results, the hydrothermal method successfully produced V_6_O_13_ cathode material with good crystallinity and consistent morphology. This material provides a reliable basis for the following study of full-cell performance.

**Figure 6:**
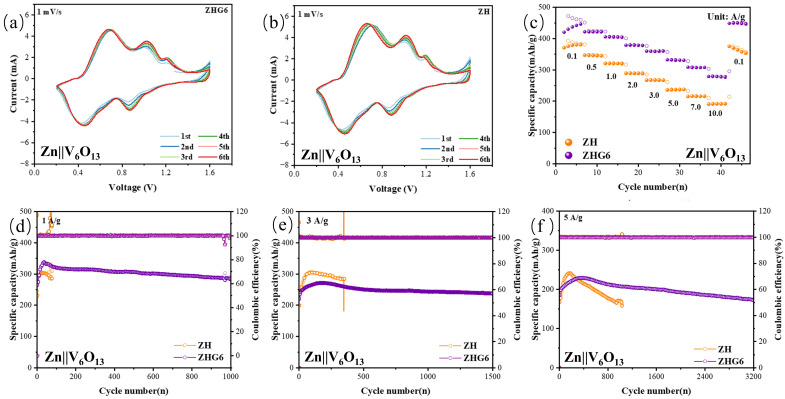
Zn||V_6_O_13_ full cells in ZH and ZHG6 electrolyte systems: (**a**) cyclic voltammetry curves of the ZHG6 system at a scan rate of 1 mV·s^−1^; (**b**) cyclic voltammetry curves of the ZH system at a scan rate of 1 mV·s^−1^; (**c**) comparison of the rate performance of the two systems at different current densities (hollow circles represent charge capacities, while the lower solid circles represent discharge capacities); (**d**–**f**) long-term cycling stability and the corresponding Coulombic efficiency of the two systems at current densities of 1 A·g^−1^, 3 A·g^−1^, and 5 A·g^−1^.

Cyclic voltammetry curves were used to analyze the redox behavior of the full cells within the voltage range of 0.2–1.6 V. The two reduction peaks located at 0.4–0.5 V and 0.7–0.8 V correspond to the insertion process of Zn^2+^ into V_6_O_13_. The two oxidation peaks located at 0.7–0.8 V and 0.9–1.0 V are related to the reversible extraction process of Zn^2+^. As shown in Figure 6a,b, the Zn||V_6_O_13_ full cell assembled with the ZHG6 electrolyte shows almost identical cyclic voltammetry curves over six cycles. This result indicates that the cathode material has good electrochemical stability and reversibility in the ZHG6 electrolyte. In contrast, the Zn||V_6_O_13_ full cell without glutaronitrile shows more obvious changes in the intensity of the redox peaks. These results suggest that the electrode reaction in the ZH system has poorer reversibility. During cycling, the polarization of the ZH-based cell may gradually increase. Its active material utilization may also decrease.

The rate performance shown in Figure 6c demonstrates the merit of the ZHG6 electrolyte. As the current density gradually increases from 0.1 to 10 A·g^−1^, the Zn||V_6_O_13_ full cell using ZHG6 always delivers a higher specific capacity than the cell using ZH. Even at a high current density of 10 A·g^−1^, the ZHG6-based cell still maintains a capacity of about 280 mAh·g^−1^. In comparison, the ZH-based cell only delivers about 190 mAh·g^−1^. When the current density returns to 0.1 A·g^−1^, the capacity of the ZHG6-based cell recovers to about 440 mAh·g^−1^. This value is close to its initial capacity. These results clearly show the excellent rate capability and electrochemical reversibility of the ZHG6-based cell.

The cycling test results confirm the advantages of the ZHG6 electrolyte. At a current density of 1 A g^−1^, the ZHG6-based cell still maintains a specific capacity of approximately 280–300 mAh g^−1^ after 1000 cycles (Figure 6d). When the current density increases to 3 A g^−1^, the cell still delivers a capacity of about 240–250 mAh g^−1^ after 1500 cycles (Figure 6e). At a higher current density of 5 A g^−1^, the cell operates stably for up to 3200 cycles and retains a reversible capacity of approximately 170–180 mAh g^−1^ (Figure 6f). The Coulombic efficiency of the ZHG6-based cell remains close to 100%. In contrast, the ZH control cell shows faster capacity decay and a shorter stable cycling period.

The improved cycling stability of the ZHG6-based full cell mainly results from the enhanced reversibility of the Zn anode. The GLN additive adjusts the Zn^2+^ solvation structure and promotes the formation of a stable ZnF_2_-containing interphase. These changes help achieve uniform Zn deposition and reduce parasitic reactions during long-term cycling.

## 4. Conclusions

In summary, glutaronitrile (GLN) was demonstrated as an effective multifunctional dinitrile additive for stabilizing Zn metal anodes in 2 M Zn(CF_3_SO_3_)_2_ aqueous electrolytes. By introducing polar C≡N groups, GLN reconstructs the local Zn^2+^ solvation environment and reorganizes the hydrogen-bonding network of water, which drastically reduces water activity, suppresses hydrogen evolution and corrosion, and improves the wettability and homogeneity of the Zn/electrolyte interface. To elucidate the regulatory mechanism, we conducted ^1^H NMR, Raman spectroscopy, contact-angle measurements, ionic-conductivity and Zn^2+^ transference-number measurements, morphological characterization, electrochemical performance evaluation, and depth-resolved interphase studies. These results reveal that the optimized ZHG6 electrolyte not only modulates Zn^2+^ coordination through Zn^2+^-N interactions and O-H···N≡C hydrogen bonding, but also promotes the formation of a chemically heterogeneous protective interphase composed of carbonaceous surface species and a ZnF_2_-enriched inner layer. As a result, ZHG6 enables compact and dendrite-suppressed Zn deposition, reduced interfacial side reactions, stable Zn||Zn cycling for over 900 h, and highly reversible Zn plating/stripping in Zn||Cu cells. Moreover, Zn||V_6_O_13_ full cells using the ZHG6 electrolyte exhibit enhanced cycling stability and rate capability, including stable operation for 3200 cycles at 5 A g^−1^. This work highlights the importance of coupling solvation-sheath reconstruction, hydrogen-bond-network regulation, and interphase chemistry through rational molecular-additive design, which provides a practical strategy for developing durable and high-performance aqueous Zn metal batteries.

## Data Availability

The original contributions presented in this study are included in the article/Supplementary Materials. Further inquiries can be directed to the corresponding authors.

## Supplementary Materials

The following supporting information can be downloaded at https://www.mdpi.com/article/10.3390/nano16150942/s1. Table S1: Comparison of representative polar electrolyte additives reported from 2023 to 2026; Figure S1: SEM images of Zn anode surfaces recovered from Zn||Zn symmetric cells after 20 cycles at 1 mA cm^−2^ and 1 mAh cm^−2^ in (a) ZH, (b) ZHG2, (c) ZHG4, (d) ZHG6, (e) ZHG8, and (f) ZHG10 electrolytes; Figure S2: Normalized ^1^H NMR spectra of the GLN methylene-proton region for GLN6 and ZHG6 at the same GLN concentration. The marked values indicate the corresponding chemical shifts; Figure S3: Normalized ^1^H NMR spectra of the water-proton region for ZH, GLN6, and ZHG6. The spectra were vertically offset for clarity; Figure S4: Ionic conductivities of the electrolytes with different GLN contents; Figure S5: Zn^2+^ transference numbers of the electrolytes with different GLN contents; Figure S6: Cross-sectional SEM image and corresponding EDS elemental mappings of the Zn electrode cycled in the ZH electrolyte: (a) cross-sectional morphology and elemental distributions of (b) O, (c) Zn, (d) F, and (e) S; Figure S7: SEM morphology of the V6O13 sample; Figure S8: XRD pattern of the synthesized V6O13 together with the standard reflections of V_6_O_13_ (JCPDS No. 27-1318).

## Abbreviations

The following abbreviations are used in this work:

AZIBs	Aqueous zinc-ion batteries
GLN	Glutaronitrile
HER	Hydrogen evolution reaction
CE	Coulombic efficiency
SEI	Solid electrolyte interphase
PVDF	Polyvinylidene fluoride
NMP	N-methyl-2-pyrrolidone
CV	Cyclic voltammetry
LSV	Linear sweep voltammetry
