# Supplementary material for: Molecular Regulation of Zn2+ Solvation Structure and Interphase Evolution by Glutaronitrile for Stable Aqueous Zinc Metal Batteries
Source: Nanomaterials (Basel). 2026 Jul 30;16(15):942. doi: 10.3390/nano16150942 (PMC13467661; doi:10.3390/nano16150942)
Supplement: Supplementary file 1 [file nanomaterials-16-00942-s001.zip › nanomaterials-4453850-supplementary.pdf]

Supplementary Information

Table S1. Comparison of representative polar electrolyte additives reported from 2023 to 2026.

Additive	Abbreviation	Testing conditions	Cycling life	References
Thioacetamid	TAA	1 mA cm^{-2} 1 mAh cm^{-2}	700 h	[42]
N-Allylthiourea	ATU	1 mA cm^{-2} 1 mAh cm^{-2}	500 h	[43]
6-aminohexanoic acid	6-AA	1 mA cm^{-2} 1 mAh cm^{-2}	800 h	[44]
epicatechin	EC	1 mA cm^{-2} 1 mAh cm^{-2}	750 h	[45]
Iminodiacetonitrile	IDAN	1.06 mA cm^{-2} 5.30 mAh cm^{-2}	440 h	[46]
1,3,6-Hexanetricarbonitrile	HTCN	5 mA cm^{-2} 5 mAh cm^{-2}	260 h	[47]
Succinic acid	SA	3 mA cm^{-2} 3 mAh cm^{-2}	650 h	[48]
Piracetam	PIR	3.51 mA cm^{-2} 3.51 mAh cm^{-2}	615 h	[49]
**Glutaronitrile**	**GLN**	**1 mA cm^{-2} 1 mAh cm^{-2}**	**905 h**	**This work**

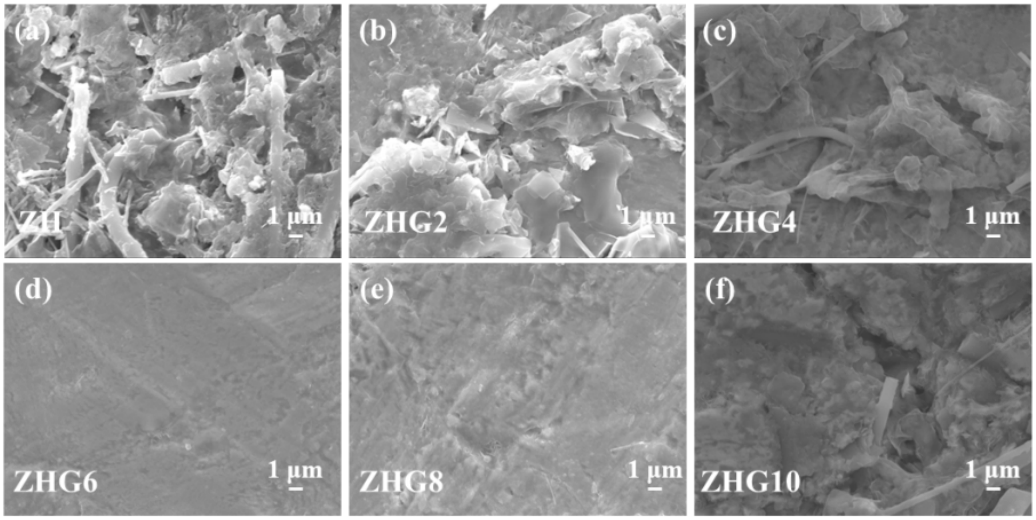

Figure S1. SEM images of Zn anode surfaces recovered from Zn||Zn symmetric cells after 20 cycles at 1 mA cm^{-2} and 1 mAh cm^{-2} in (a) ZH, (b) ZHG2, (c) ZHG4, (d) ZHG6, (e) ZHG8, and (f) ZHG10 electrolytes.

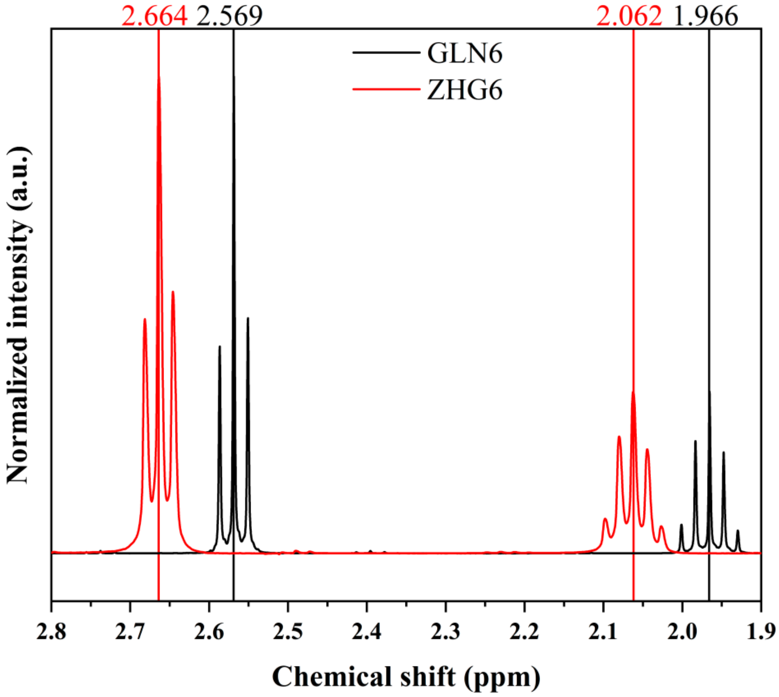

Figure S2. Normalized ^{1}H NMR spectra of the GLN methylene-proton region for GLN6 and ZHG6 at the same GLN concentration. The marked values indicate the corresponding chemical shifts.

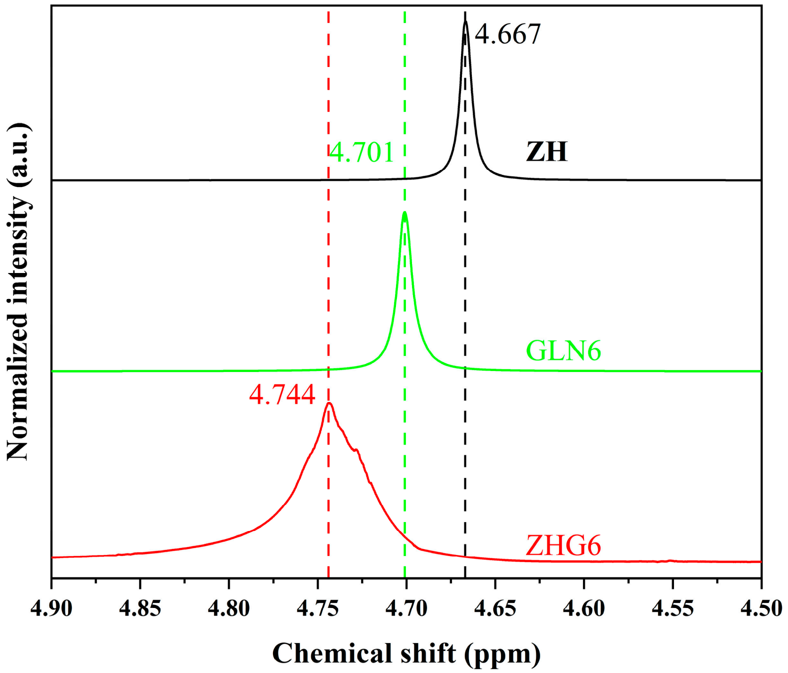

Figure S3. Normalized ^{1}H NMR spectra of the water-proton region for ZH, GLN6, and ZHG6. The spectra were vertically offset for clarity.

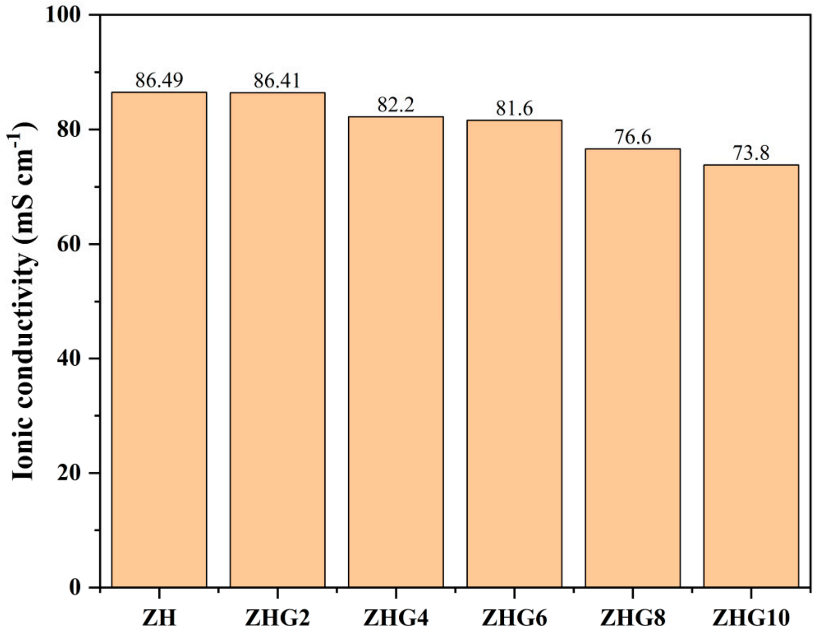

Figure S4. Ionic conductivities of the electrolytes with different GLN contents.

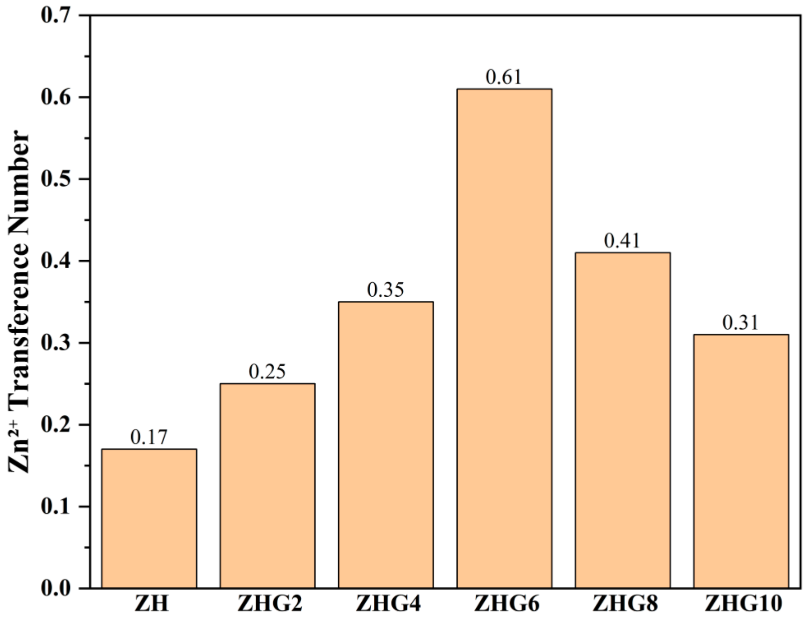

Figure S5. Zn^{2+} transference numbers of the electrolytes with different GLN contents.

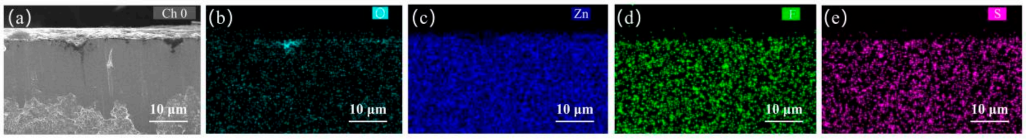

Figure S6. Cross-sectional SEM image and corresponding EDS elemental mappings of the Zn electrode cycled in the ZH electrolyte: (a) cross-sectional morphology and elemental distributions of (b) O, (c) Zn, (d) F, and (e) S.

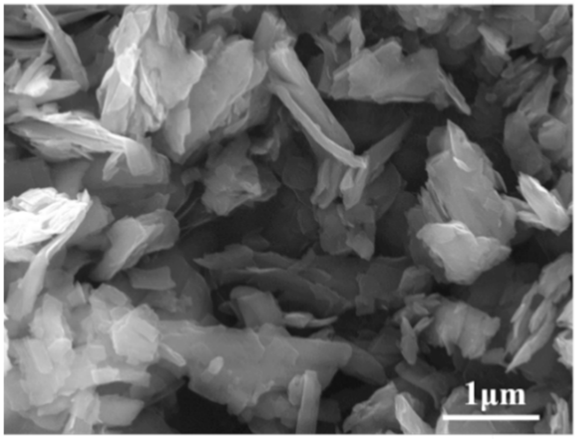

Figure S7. SEM morphology of V_6O_{13} sample.

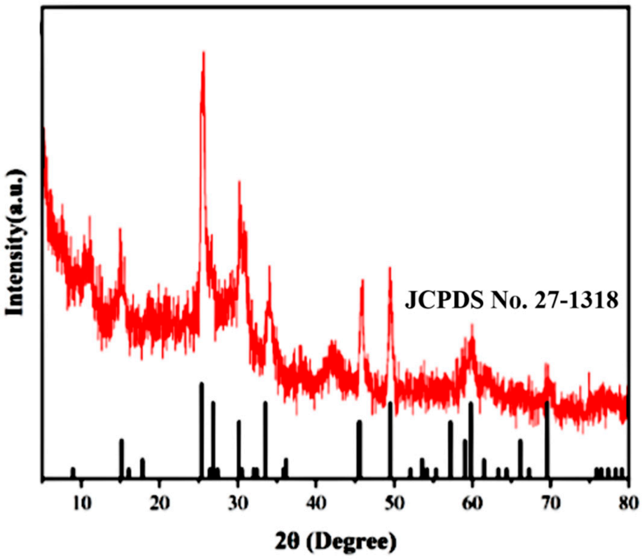

Figure S8. XRD pattern of the synthesized V_6O_{13} together with the standard reflections of V_6O_{13} (JCPDS No. 27-1318).
